# TRPC absence induces pro-inflammatory macrophage polarization to promote obesity and exacerbate colorectal cancer

**DOI:** 10.3389/fphar.2024.1392328

**Published:** 2024-05-21

**Authors:** Yanting Lin, Rui Gao, Dongquan Jing, Yiming Liu, Huijuan Da, Lutz Birnbaumer, Yong Yang, Xinghua Gao, Zhenhua Gao, Qiuhua Cao

**Affiliations:** ^1^ Center for New Drug Safety Evaluation and Research, State Key Laboratory of Natural Medicines, China Pharmaceutical University, Nanjing, Jiangsu, China; ^2^ Institute of Biomedical Research (BIOMED), Catholic University of Argentina, Buenos Aires, Argentina; ^3^ Signal Transduction Laboratory, National Institute of Environmental Health Sciences, Research Triangle Park, NC, United States; ^4^ Shandong University Cancer Center, Jinan, Shandong, China; ^5^ Department of Radiation Oncology, Shandong Cancer Hospital and Institute, Shandong First Medical University and Shandong Academy of Medical Sciences, Jinan, Shandong, China; ^6^ Vaccine Center, School of Basic Medicine and Clinical Pharmacy, China Pharmaceutical University, Nanjing, Jiangsu, China

**Keywords:** TRPC HeptaKO, macrophages, obesity, gut microbiota, colorectal cancer

## Abstract

During the past half-century, although numerous interventions for obesity have arisen, the condition’s prevalence has relentlessly escalated annually. Obesity represents a substantial public health challenge, especially due to its robust correlation with co-morbidities, such as colorectal cancer (CRC), which often thrives in an inflammatory tumor milieu. Of note, individuals with obesity commonly present with calcium and vitamin D insufficiencies. Transient receptor potential canonical (TRPC) channels, a subclass within the broader TRP family, function as critical calcium transporters in calcium-mediated signaling pathways. However, the exact role of TRPC channels in both obesity and CRC pathogenesis remains poorly understood. This study set out to elucidate the part played by TRPC channels in obesity and CRC development using a mouse model lacking all seven TRPC proteins (TRPC HeptaKO mice). Relative to wild-type counterparts, TRPC HeptaKO mice manifested severe obesity, evidenced by significantly heightened body weights, augmented weights of epididymal white adipose tissue (eWAT) and inguinal white adipose tissue (iWAT), increased hepatic lipid deposition, and raised serum levels of total cholesterol (T-CHO) and low-density lipoprotein cholesterol (LDL-C). Moreover, TRPC deficiency was accompanied by an decrease in thermogenic molecules like PGC1-α and UCP1, alongside a upsurge in inflammatory factors within adipose tissue. Mechanistically, it was revealed that pro-inflammatory factors originating from inflammatory macrophages in adipose tissue triggered lipid accumulation and exacerbated obesity-related phenotypes. Intriguingly, considering the well-established connection between obesity and disrupted gut microbiota balance, substantial changes in the gut microbiota composition were detected in TRPC HeptaKO mice, contributing to CRC development. This study provides valuable insights into the role and underlying mechanisms of TRPC deficiency in obesity and its related complication, CRC. Our findings offer a theoretical foundation for the prevention of adverse effects associated with TRPC inhibitors, potentially leading to new therapeutic strategies for obesity and CRC prevention.

## 1 Introduction

Obesity, characterized by an excessive accumulation of body fat to the extent that it impairs health, is a pervasive chronic metabolic disorder driven by a complex interplay of genetic, hormonal, and environmental influences. In 2022, the prevalence of obesity among the pediatric and adolescent population soared to levels fourfold higher than those observed in 1990. Concurrently, the incidence of obesity in adults witnessed a stark escalation, with rates more than doubling among women and nearly tripling among men. This alarming trend culminates in a staggering 159 million young individuals and 879 million adults grappling with the condition, foreshadowing a cascade of associated complications and a consequential surge in healthcare expenditures (NCD Risk Factor Collaboration NCD-RisC, 2024). Obesity’s impact extends beyond the heightened risk of traditional comorbidities such as fatty liver, diabetes, and hypertension; it also plays a significant role in the etiology of certain cancers that thrive in an inflammatory tumor microenvironment (Bluher, 2019). Notably, colorectal cancer (CRC), the third most common malignant neoplasm, exhibits a pronounced correlation with obesity (Wunderlich et al., 2018), with the obesity-altered gut microbiota emerging as a pivotal factor in the pathogenesis of CRC (Beyaz et al., 2021; Yang et al., 2022).

The onset of obesity is characterized by a dysfunctional expansion of white adipose tissue (WAT) and impaired functionality of brown adipose tissue (BAT), a phenomenon often referred to as “BAT whitening” (Cairo et al., 2016). The lipid accumulation observed in obesity is intricately linked to adiponectin secretion levels; elevated adiponectin levels are associated with a diminished risk of metabolic complications arising from obesity, while diminished levels of adiponectin are correlated with an increased susceptibility to metabolic irregularities, hypertension, and hypertriglyceridemia (Achari and Jain, 2017; Zhao et al., 2021). And ω-3 fatty acids are known to augment the secretion of adiponectin, an adipokine with anti-inflammatory properties (Sukumar et al., 2012). Notably, study has highlighted a significant relationship between adiponectin secretion by adipocytes and the activity of calcium channels, with certain channels exerting an inhibitory effect on adiponectin production. Morover, obesity is frequently coupled with deficiencies in calcium and vitamin D, which can exacerbate the condition (Vrieling and Stienstra, 2023). Transient receptor potential canonical (TRPC) channels are a class of calcium transport channels that belong to the larger TRP superfamily, specifically a subset known as receptor-operated calcium-permeable nonselective cation channels. The seven known mammalian TRPC proteins can be categorized into four distinct subgroups—TRPC1, TRPC2, TRPC4/5, and TRPC3/6/7—based on their amino acid sequences and functional attributes, and they partake in a diverse array of cellular processes and physiological functions (Wang et al., 2020). Although there are some reports on the role of the TRPC family in obesity, the specific role of channels in the family in obesity is unclear.

It has been reported that TRPC1 and TRPC5 are significantly upregulated in mature adipose tissue, leading to an efflux of Ca^2+^ and subsequently inhibiting the production of adiponectin, a crucial adipokine beneficial to the cardiovascular system (Sukumar et al., 2012). Consistent with this finding, another study suggests that the absence of TRPC1 promotes obesity induced by a high-fat diet, and downregulation of TRPC1 is associated with a distinct metabolic phenotype as well as significant changes in the expression of key BAT markers (Wolfrum et al., 2018). Additionally, the TRPC3 channel enhances sensitivity in gustatory perception of dietary lipids, potentially influencing preferences for lipid intake and thereby affecting the occurrence of obesity (Murtaza et al., 2021). On the other hand, TRPC4/TRPC5 has been shown to protect against hyperglycemia and dyslipidemia caused by a high-sucrose diet (Araujo et al., 2023). However, interestingly, deletion of TRPC5 in the central amygdala has been shown to counteract obesity induced by a high-fat diet through an increase in sympathetic innervation (Ma et al., 2022). As a result, the role of TRPC channels in obesity remains controversial. Obesity is known to be associated with a wide range of complications, CRC being one of the primary ones. Research indicates that obesity can lead to gut microbiota disorders or promote intestinal stem cell proliferation, thereby increasing the risk of CRC (Fu et al., 2019; Yang et al., 2022). However, the question of whether obesity induced by TRPC deletion promotes CRC development remains unanswered and requires further investigation. In conclusion, multiple TRPC channels are implicated in the progression of obesity and obesity-related complications, yet the underlying mechanisms remain elusive.

As we previously described (Lin et al., 2023), TRPC inhibitors have garnered widespread attention in clinical and preclinical settings as potential therapeutics for a range of diseases, including cardiovascular ailments and diabetic nephropathy (Liu et al., 2024). These inhibitors represent promising drug candidates, exhibiting diverse therapeutic potentials. However, there remains a paucity of information regarding their potential adverse effects, particularly in the context of obesity and obesity-driven colorectal cancer (CRC). Notably, many current TRPC inhibitors lack specificity, affecting multiple TRPC channels simultaneously (Miller et al., 2011; Zhu et al., 2015; Yu et al., 2019), thus posing significant challenges in elucidating the precise role of these channels in obesity and obesity-related diseases. To address this gap, our current research employed mice lacking all seven TRPC proteins (TRPC HeptaKO mice) to comprehensively investigate the role of TRPC channels in obesity and its related complication, CRC. Our findings revealed that TRPC deficiency led to an increase in pro-inflammatory macrophages and inflammatory factors within adipose tissue, promoting lipid accumulation and exacerbating obesity. Furthermore, this TRPC-deficiency-induced obesity significantly disrupted the gut microbiota, thereby accelerating CRC progression. Collectively, this study elucidates the role and mechanism of TRPC deficiency in obesity and related complications of CRC and provides a potential theoretical basis for the prevention of adverse effects of TRPC inhibitors.

## 2 Materials and methods

### 2.1 Ethics statement

All experiments involving animals were conducted according to the ethical policies and procedures approved by the ethics committee of the Center for New Drug Safety Evaluation and Research, China Pharmaceutical University (Approval no. B20190624-2).

### 2.2 Mice

The TRPC Hepta control (wild-type, WT, 50% 129SvEv:50% C57BL/6J intercrossed >6 times) and TRPC-hepta knockout (TRPC HeptaKO) mice lacking all seven TRPC proteins were originally created under the guidance of Lutz Birnbaumer at the Comparative Medicine Branch of the National Institute of Environmental Health Sciences, Research Triangle Park, USA, by combining single KO alleles (Formoso et al., 2020), which supplied them to us. Male mice were weighed from 21 days old until about 2 months old and dissected. Lee’s index = weight (g)^(1/3) x 10/body length (cm), where body length is defined as the distance from the nose to the anus (Bernardis and Patterson, 1968; Cheng et al., 2023).

WT and TRPC HeptaKO breeding mice were fed Experimental Rodent Growth and Reproduction Diet (Product No. 1010002, supplied by Jiangsu Synchronized Pharmaceutical & Biological Engineering Co., Ltd.), with primary ingredients consisting of imported fishmeal, chicken meat, soybean meal, northeastern corn, wheat, soybean oil, alfalfa hay, bran, a variety of amino acids, vitamins, and other components. After the mice were separated into individual cages, they were provided with Experimental Rodent Maintenance Diet (Product No. kawc3211, manufactured by Keao Xieli Tianjin Feed Co., Ltd.), which mainly comprised corn, soybean meal, flour, bran, dicalcium phosphate, fishmeal, limestone powder, sodium chloride, vitamin premixes, and trace mineral premixes, among others. Mice were housed in the Center for New Drug Safety Evaluation and Research, China Pharmaceutical University.

### 2.3 Histological analysis and immunohistochemistry

For histological analysis, adipose and colon tissues underwent fixation in 4% paraformaldehyde for a period of 1 day. Subsequently, 4-μm paraffin-embedded sections were stained with Hematoxylin and eosin (H&E) to evaluate tissue morphology and the infiltration of inflammatory cells. The CRC pathological scoring method was modified based on previously reported (Cao et al., 2024). In brief, the scoring system ranges from 0 to 4, with 0 representing normal tissue, one indicating mild inflammation or epithelial loss, two reflecting moderate damage including elongated crypts with hyperchromatic epithelium, loss of crypt branching, slightly enlarged and crowded cell nuclei, and marginally reduced intracellular mucin, three represents marked damage with crypts twice or three times the normal thickness, hyperchromatic epithelium, reduced goblet cells, and scattered arborization, and four denotes severe damage with crypts twice or three times the normal thickness, marked hyperchromasia, few or no goblet cells, a high mitotic index, and frequent arborization. For each adipose tissue section, three representative fields were selected for photography, and the number of adipocytes and adipose areas in each field were counted using ImageJ. software.

For immunohistochemistry, the tissue slides were incubated overnight at 4°C with specific anti-PGC-1α antibody (1:150, Cat No. 66369-1-Ig, Proteintech, China), anti-UCP1 antibody (1:200, Cat No. 23673-1-AP, Proteintech, China) and anti-proliferating cell nuclear antigen (PCNA) antibody (1:2000, Cat No. #2586, CST, USA), then the sections were incubated with HRP secondary antibodies (Cat No. KIT-5002 or KIT-5005, MXB Biotechnologies, China) to enhance the signal. The colonic sections were visualized using a Diaminobenzidine Substrate Kit (Cat No. DAB-1031, MXB Biotechnologies, China) and counterstained with hematoxylin. The sections were photographed using a microscope (NE900; Nexcope, China). For each tissue section, three representative fields were selected for photography. These fields should be evenly distributed throughout the section to capture the overall picture of positive cells throughout the section. The number of positive cells was counted by ImageJ. software. For each tissue section, three representative fields were selected for photography. These fields should be evenly distributed throughout the section to capture the overall picture of positive cells throughout the section. The number of positive cells was counted by ImageJ. software.

### 2.4 Oil red O staining

The tissues were incubated in optimal cutting temperature compound (OCT) and sliced into 10 μm slides. The slides were stained with oil-red O working solution at room temperature for 30 min. Images were acquired by microscope. The lipid droplet area was measured with ImageJ. software.

### 2.5 Serum lipid assay

Lipid-associated molecules (T-CHO, TG, LDL-C, HDL-C) present in the serum were assayed in accordance with the manufactures’s instructions (Cat No. A111-1/A110-1/A113-1/A112-1, Nanjing Jiancheng Bioengineering Institute, China). Briefly, 250 μL of working solution and 2.5 μL of serum sample were added to each well of a 96-well plate. After mixing, the samples were incubated at 37°C for 10 min. The absorbance value of each well was measured at 510 nm or 500 nm using a microplate reader. Finally, the concentration of T-CHO/TG in serum was calculated according to the formula: T-CHO/TG (mmol/L) = ((Sample OD value - Blank OD value)/(Calibration OD value - Blank OD)) × Concentration of calibrator.

### 2.6 Quantitative Real-Time PCR

Total RNA was extracted using the FastPure Cell/Tissue Total RNA Isolation Kit (Cat No. RC101-01, Vazyme, China). cDNA synthesis was executed with a cDNA synthesis kit (Cat No. #RR047A, Takara, China). RT-PCR reactions were performed by Step one plus Real-Time PCR system (Applied Biosystems, USA), employing the ChamQ™ SYBR qPCR Master Mix (Cat No. Q341-02, Vazyme, China). The results of PCR were calculated by the 2^△△CT^ method and normalized to *β-Actin*. All primers were synthesized at Genscript (Nanjing, China). The primer sequences were shown in the Supplementary Table 1.

### 2.7 Flow cytometry

The immune cells were isolated from both epididymal white adipose tissue (eWAT) and inguinal white adipose tissue (iWAT) according to previously protocol (Anderson-Baucum et al., 2021). Briefly, the adipose tissue was sheared on ice and digested with a collagenase digestive solution (0.5% bovine serum albumin (BSA) + 10 mM CaCl2 + 4 mg/mL collagenase II (Cat No. C6885, Sigma-Aldrich) for 20 min in a shaker at 37°C. The isolated cells were resuspended in FACS buffer (0.5% BSA in Dulbeccos phosphate buffered saline (DPBS)) and were passed through a 70-μm strainer. Samples were analyzed with following antibodies: Live-dead-NIR (Cat No. #L34976, Thermo Fisher Scientific, USA), CD45-FITC (Cat No. #103108, Biolegend, USA), CD11b-SB702 (Cat No. #67-0112-82, ebioscience, USA), F4/80-APC (Cat No. #17-4801-82, ebioscience, USA), CD11c-BV421 (Cat No. #117330, Biolegend, USA). Data were acquired on Attune NxT flow cytometer (Thermo Fisher Scientific, USA) and data were analyzed with FlowJo software.

### 2.8 Isolation of bone marrow-derived macrophages (BMDMs)

Extraction of BMDMs from the femur and tibia of mice was performed as previously described (Miao et al., 2014). Briefly, BMDMs were collected from 6-8-week-old male mice by flushing the femur and tibia of mice with cold cold phosphate-buffered saline (PBS). BMDMs were cultured in DMEM containing 10% heat-inactivated FBS, 1% Penicillin-streptomycin Solution and 10 ng/mL of mouse M-CSF (Cat No. 315-05, Peprotech, USA) for 6–7 days, at 37°C with 5% CO_2_.

### 2.9 Macrophage polarization

BMDMs were treated with 20 ng/mL IFN-γ (Cat No.315-05, Peprotech, USA) and 100 ng/mL LPS (Cat No. L6529, Sigma, USA) for 6 h or 24 h to induce M1 macrophages. 20 ng/mL IL-4 (Cat No.214-14, Peprotech, USA) were used to induce M2 macrophages.

### 2.10 Cytokine measurement

Conditioned supernatants from BMDMs were collected and measured for the levels of IL-6 (Cat No. DY406-05, R&D Systems, USA), TNF-α (Cat No. DY410-05, R&D Systems, USA), and IL-12 p70 (Cat No. DY419-05, R&D Systems, USA).

### 2.11 Periodic acid-Schiff (PAS) staining

PAS staining was used to assess the number of goblet cells. For PAS staining, the colonic sections were incubated with the periodic acid solution (Cat No. BP-DL031; SenBeiJia BioTech CO., Ltd., China) for 5–8 min after the sections had been dewaxed and hydrated. Then, the colonic sections were incubated with Schiff reagent for 20 min and counterstained with hematoxylin. The colon sections were photographed using a microscope (NE900; Nexcope, China). Five microscopic fields (400×) from different areas of each colonic section were used for counting positive cells.

### 2.12 16S rRNA gene sequencing and analysis

The DNA was extracted from colon contents. DNA library preparation and 16S ribosomal RNA gene sequencing were performed by NovoGene (Tianjin, China). Amplification of the V3-V4 regions of 16S rRNA genes were amplified using specific primers (341F [CCTAYGGGRBGCASCAG] and 806R [GGACTACNNGGGTATCTAAT]). The 16S rRNA gene sequence data were quality-filtered and analyzed using QIIME2 software. The sequencing errors and replicated sequences were detected by Deblur algorithm. Before dereplicating sequences that encoded the amplicon sequence variants (ASV), paired reads were joined and trimmed to 404 base pairs. After filtering chimera sequences, the dereplicated sequences were classified taxonomically using Greengenes 16S rRNA gene reference database at a 99% identity cut-off by VSEARCH software. Beta diversity was measured by Arrhenius z distance, and Principal Coordinates Analysis (PCoA) was used for ordination analysis. Community dissimilarities were tested by permutational multivariate analyses of variance (PERMANOVA) with 1,000 iterations.

### 2.13 Antibiotic cocktail (Abx) experiment

Mice drunk water solution containing 1 g/L ampicillin (Cat No. A105483, Aladdin, China), 1 g/L neomycin (Cat No. N109017, Aladdin, China), 0.5 g/L metronidazole (Cat No. M109874, Aladdin, China), and 0.5 g/L vancomycin (Cat No. V105495, Aladdin, China) through the experiment procedure.

### 2.14 Azoxymethane/dextran sulfate sodium (AOM/DSS)-induced CRC model

AOM/DSS-induced CRC model was performed according to previously protocol (Grivennikov et al., 2009). On day 1, the age-and-sex-matched WT and TRPC HeptaKO mice were injected i. p. with 10 mg/kg azoxymethane (AOM) (Cat No. A5486, Sigma-Aldrich, USA). After 7 days, mice were treated with 1% dextran sulfate sodium (DSS, M.W. 36000–50000 Da, MP Biomedical, Canada) for 1 week and treated with regular water for another 2 weeks. After that, mice were subjected to two more DSS treatment cycles.

### 2.15 Statistical analysis

GraphPad Prism 8.3.0 was used for all statistical analyses. Data are presented as the mean ± SEM. When the variances of the two groups are homogeneous, we employed parametric tests using the Student’s t-test. However, when the variances between the two groups are not equal, we resorted to non-parametric testing (Mann-Whitney U test) to confirm the normal distribution of data. One-way ANOVA was used when more than two groups were compared, and two-way ANOVA was used when data were compared based on curves. Survival rates were evaluated using the Kaplan−Meier survival method. *p* < 0.05 was considered statistically significant.

### 2.16 Data availability

All data relevant to the study are included in the article or have been uploaded as supplementary information. All other data supporting the findings of this study are available from the corresponding author upon reasonable request.

## 3 Results

### 3.1 Lacking all seven TRPC proteins induces obesity in mice

**FIGURE 1:**
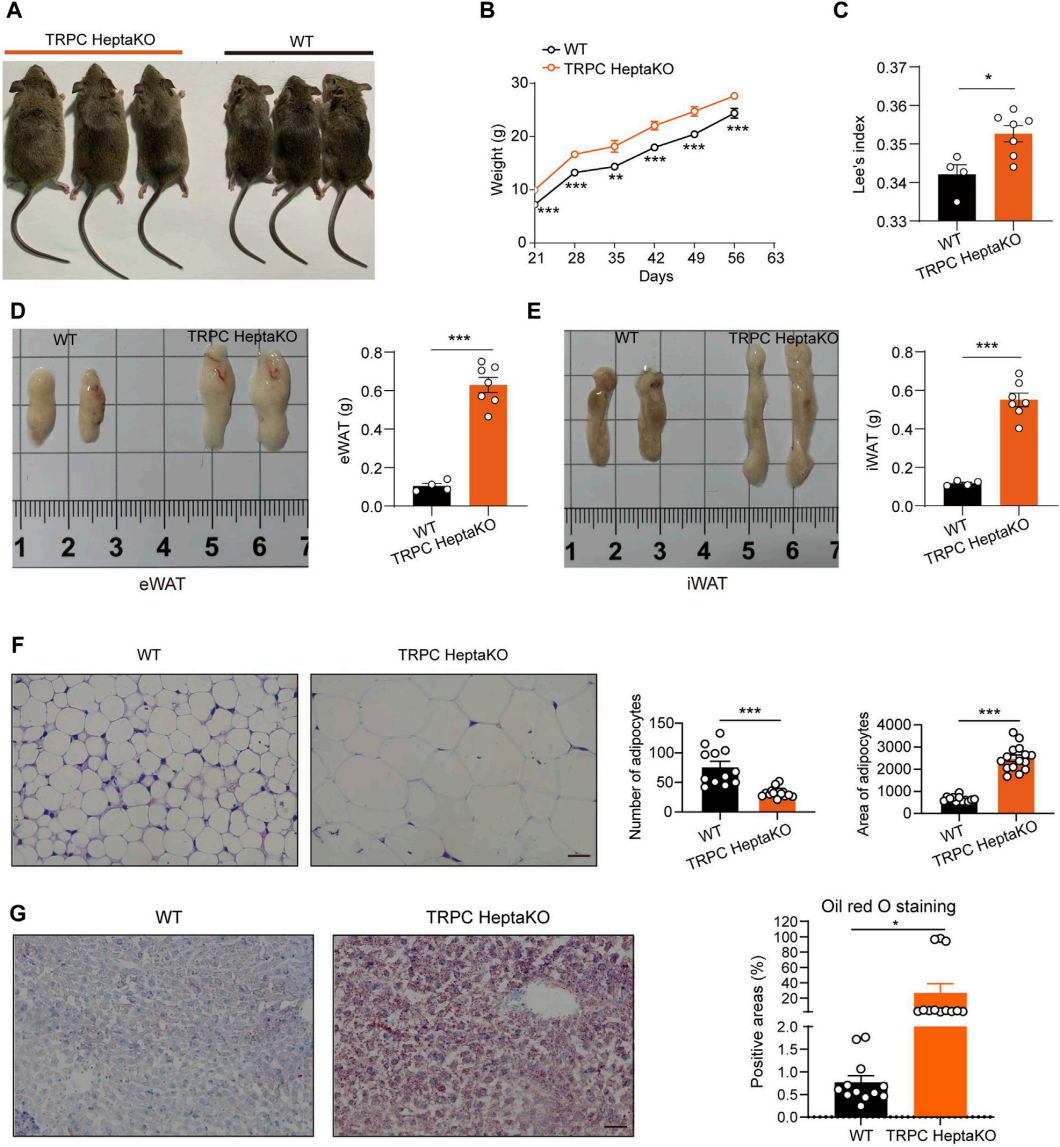
Ablation all seven TRPC proteins induces obesity in mice. **(A)** Representative pictures of 8-week-old WT and TRPC HeptaKO mice. **(B)** Body weight of WT and TRPC HeptaKO mice (n = 7–8). **(C)** Lee’s index of WT and TRPC HeptaKO mice (n = 4–7). **(D)** Anatomical pictures of eWAT of WT and Hepta-KO mice (Left). Weight of eWAT in WT and TRPC HeptaKO mice (n = 4–7) (Right). **(E)** Anatomical pictures of iWAT of WT and HeptaKO mice (Left). Weight of iWAT in WT and TRPC HeptaKO mice (n = 4–7) (Right). **(F)** Representative images and statistical graphs of H&E staining in the fixed eWAT (n = 4–5, three fields/slice). Scale bar: 50 μm. **(G)** Representative pictures and statistical graphs in Oil red-stained liver sections (n = 4, three fields/slice). The scale bar, 50 μm. In Figure 1B, Two-way ANOVA test was used, whereas for the other figures, Two-tailed Student’s t-test was employed. *p < 0.05, ***p < 0.001.

During the cultivation of TRPC-deficient mice, our observations revealed that the TRPC HeptaKO mice exhibited a pronounced increase in adiposity (Figure 1A), consistent weight gain (Figure 1B), and an elevated Lee’s index (Figure 1C) in comparison to their WT counterparts. These findings indicate that the absence of all seven TRPC proteins markedly accelerates the onset of obesity in mice. A hallmark of obesity is the excessive accumulation of adipose tissue, and we observed that TRPC HeptaKO mice displayed a significant upsurge in the weight of both eWAT and iWAT relative to WT mice (Figures 1D,E). Furthermore, histological analysis through H&E staining disclosed a substantial enlargement of adipocyte volume in the eWAT of TRPC HeptaKO mice (Figure 1F). Given the propensity for lipid disorders in the liver of obese individuals, we conducted oil red O staining on liver tissue sections, which confirmed that TRPC deficiency markedly enhanced hepatic lipid accumulation (Figure 1G). Collectively, these results imply that TRPC channels may act as regulatory brakes against obesity development.

**FIGURE 2:**
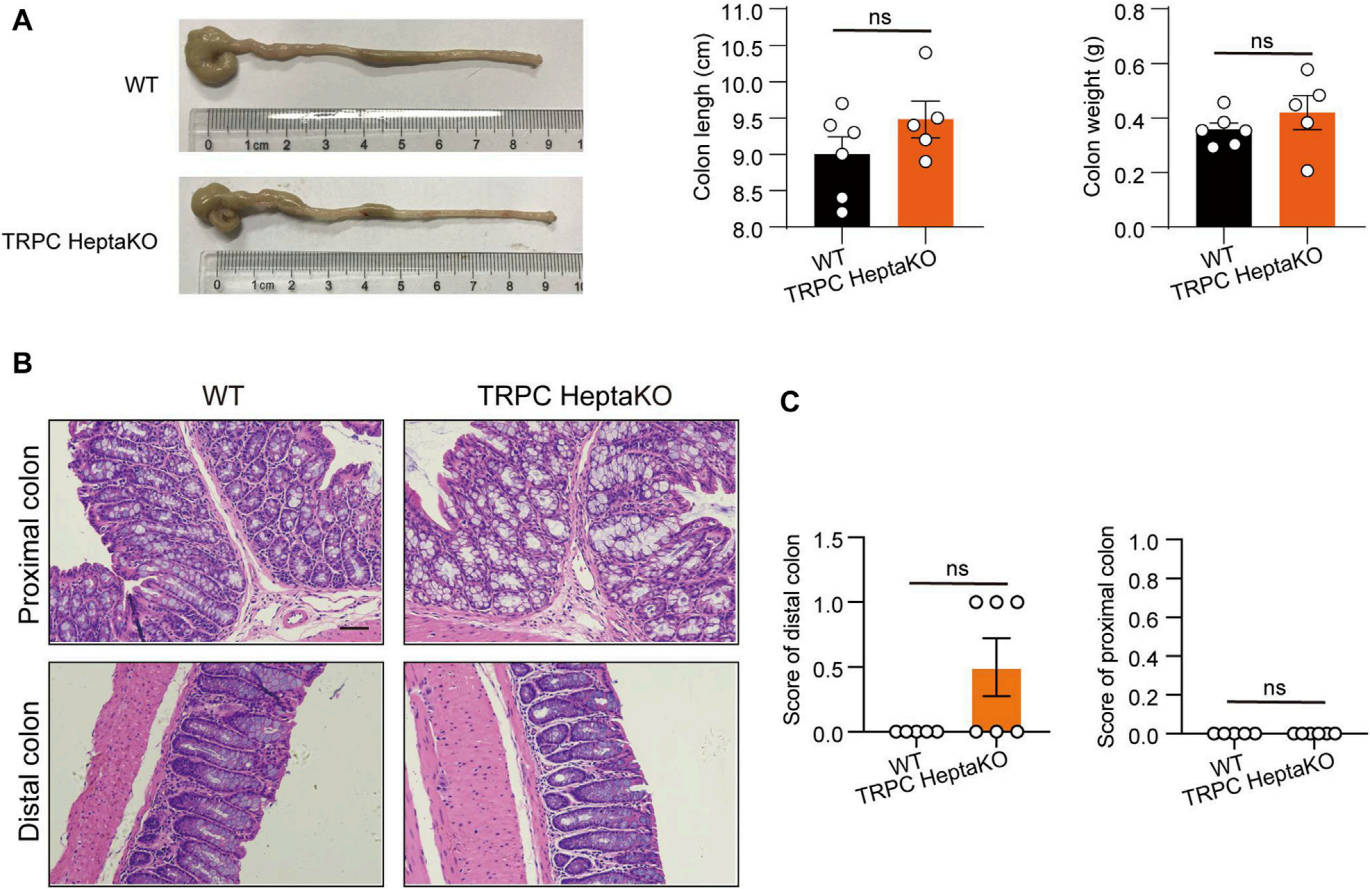
TRPC absence has no significant effects on colon tissues. **(A)** Representative colon images of WT and TRPC HeptaKO mice (Left). Colon length and colon weight (Right) (n **=** 6). **(B, C)** Representative microscopic images in H&E staining and pathological scores in the colon tissues. Scale bar: 50 μm. (n = 5–6). Two-tailed Student’s t-test was used to determine statistical significance between two groups. ns, no significant.

Building on our previous findings that TRPC deficiency can induce pro-inflammatory macrophages and disrupt gut microbiota, thereby increasing susceptibility to colitis (Lin et al., 2023), we dissected the colons of both WT and TRPC-deficient mice. Our analysis showed that the absence of TRPC did not influence the colon’s length or weight (Figure 2A). Moreover, H&E staining indicated that the absence of TRPC did not significantly affect the histological integrity or the pathological score of colonic tissues under physiological conditions (Figures 2B,C). This suggests that the obesity induced by TRPC deletion is not inherently linked to structural alterations in the colonic tissue.

### 3.2 TRPC absence inhibits adipose tissue thermogenesis

**FIGURE 3:**
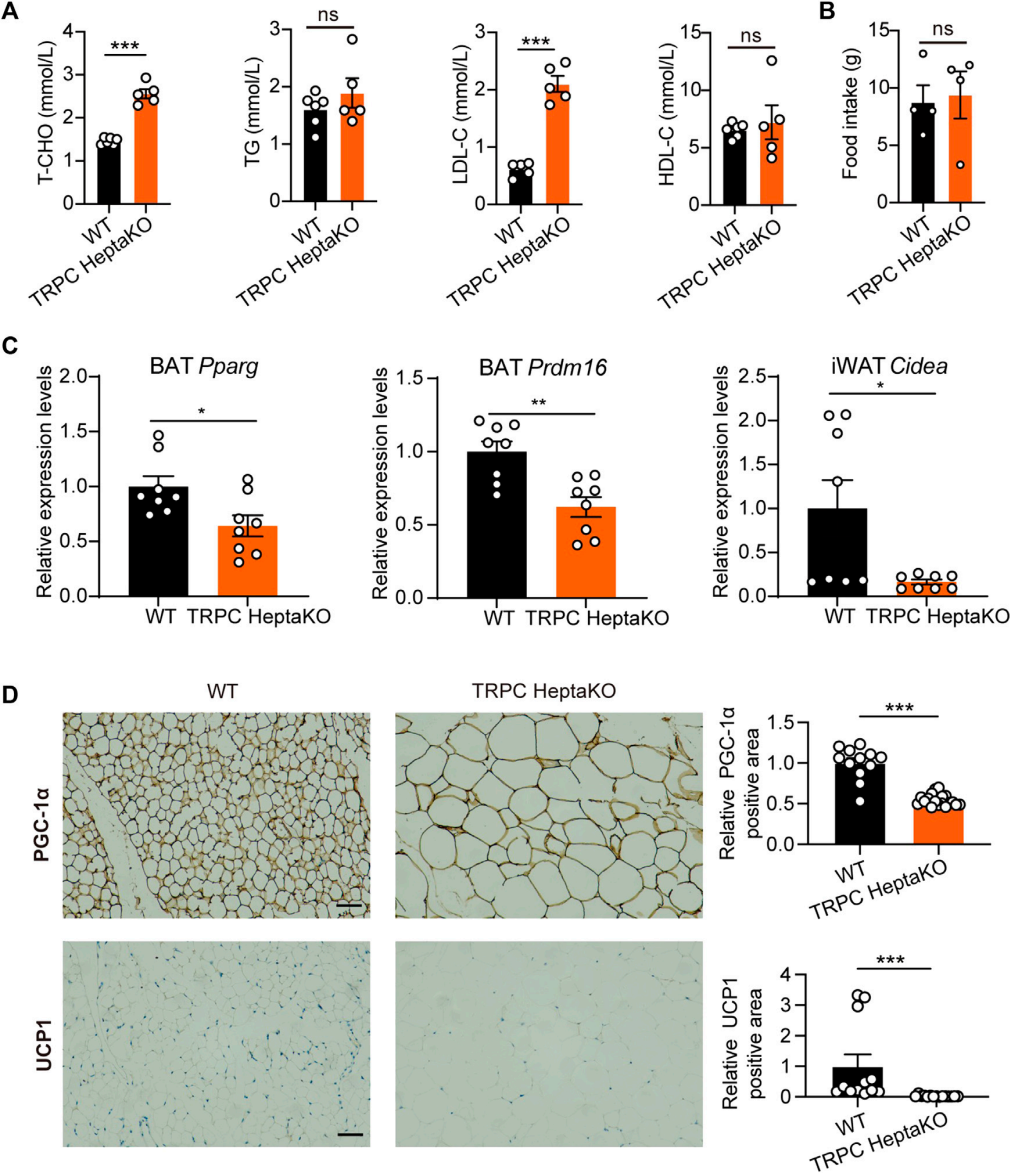
TRPC absence inhibits adipose tissue thermogenesis. **(A)** The serum T-CHO, TG, LDL-C, HDL-C levels in WT and TRPC HeptaKO mice (n = 5–6). **(B)** Average food intake per three mice for 24 h (n = 4). **(C)** Fatty acid regulation genes in adipose tissues of mice (n = 8). **(D)** Representative immunohistochemistry images showing PGC-1α and UCP1 in eWAT (n = 4–6, three fields/slice), Scale bar = 50 μm. Two-tailed Student’s t-test was used to determine statistical significance between two groups. *p < 0.05, **p < 0.01, ***p < 0.001, ns, no significant.

Recent investigations have underscored a connection between obesity and metabolic dyslipidemia, which is typified by elevated serum concentrations of low-density cholesterol (LDL-C), triglyceride (TG), and total cholesterol (T-CHO), alongside diminished levels of high-density cholesterol (HDL-C) (Howard et al., 2003; Ginsberg et al., 2006; Kathiresan et al., 2006; Rader, 2007; Curley et al., 2021). In our study, the TRPC HeptaKO mice demonstrated a significant increase in both T-CHO and LDL-C levels compared to their WT counterparts (Figure 3A). Notably, the deficiency in TRPC did not affect the mice’s food consumption patterns (Figure 3B). Thus, we concluded that TRPC HeptaKO mice were obese and associated with dyslipidemia. Previous research has indicated that the expression of critical thermogenic genes is suppressed in individuals with obesity (Yan et al., 2021). In the present research, we systematically assessed a suite of thermogenic genes, revealing that the deletion of TRPC led to a significant reduction in the mRNA expression levels of *Pparg* and *Prdm16* in BAT (Figure 3C). Concurrently, we observed that *Cidea* mRNA expression was similarly downregulated in TRPC-deficient iWAT (Figure 3C). Furthermore, immunohistochemical staining evidenced a decrease in the protein levels of PGC1-α and UCP1 in TRPC-deficient eWAT (Figure 3D). The above results suggest that the deficiency of TRPC impairs lipid metabolism and concurrently suppresses adipose tissue thermogenesis, which appears to be a critical contributing factor to the development and progression of obesity.

### 3.3 TRPC deficiency promotes macrophage accumulation and elevates inflammatory factors in adipose tissue

**FIGURE 4:**
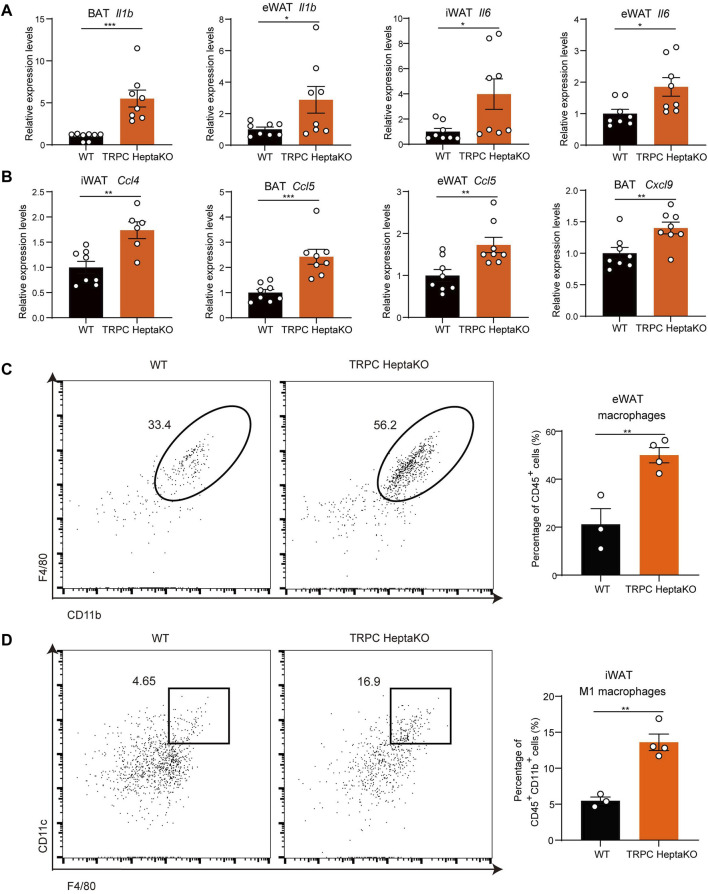
TRPC deficiency increases macrophages and inflammatory factors in adipose tissue. **(A, B)** RT‒PCR results of inflammatory factors and chemokines in adipose tissues of mice (n = 6–8). **(C)** Live CD45^+^ CD11b^+^ F4/80^+^ macrophages in eWAT of mice were identified by flow cytometry. Left: the representative images; right: the statistical graph of macrophages (n = 3–4). **(D)** Live CD45^+^ CD11b^+^ F4/80^+^ CD11c^+^ macrophages (M1 macrophages) in iWAT of mice were identified by flow cytometry. Left: the representative images; right: the statistical graph of macrophages (n = 3–4). Two-tailed Student’s t-test was used to determine statistical significance between two groups. *p < 0.05, **p < 0.01, ***p < 0.001.

Persistent low-grade inflammation, predominantly driven by aberrant immune cell activation within adipose tissue, represents a crucial pathophysiological characteristic in adipose tissue dysfunction. Among these immune cells, the role of macrophages stands out as particularly influential, particularly concerning the recruitment and activation of pro-inflammatory macrophages (Lumeng et al., 2007; Chavakis et al., 2023). Studies have shown that in both obese mice and humans, there is a pronounced increase in the proportion of M1-type (pro-inflammatory) macrophages, paralleled by escalated levels of inflammatory mediators such as TNF-α, IL-1, IL-6, and additional inflammatory factors (Lu et al., 2023). It has been shown that diminishing the population of M1-type (pro-inflammatory) macrophages or facilitating their transition to anti-inflammatory M2-type macrophages can mitigate obesity progression and lower the risk of long-term complications. Therefore, the involvement of macrophages in obesity and its related comorbidities is of paramount importance. In this research, we found TRPC absence increased the mRNA levels of *Il1b* and *Il6* in adipose tissues (Figure 4A). Given that adipose macrophages are a major source of secreted inflammatory factors, we examined macrophage chemokines and macrophage numbers and showed that TRPC deletion significantly promoted the levels of macrophage chemokines *Ccl4*, *Ccl5*, and *Cxcl9* in adipose tissue (Figure 4B). Moreover, we also ascertained that TRPC depletion induced a significant rise in the number of macrophages in eWAT (Figure 4C), along with an increase in the count of inflammatory macrophages within iWAT as confirmed by flow cytometry analysis (Figure 4D). Altogether, these results suggest that TRPC absence substantially escalates macrophage infiltration and related inflammatory cytokines, which likely play a pivotal role in impairing adipocyte thermogenesis and thereby exacerbating obesity.

### 3.4 TRPC HeptaKO macrophages exhibit enhanced M1 polarization and augmented proinflammatory cytokine secretion

**FIGURE 5:**
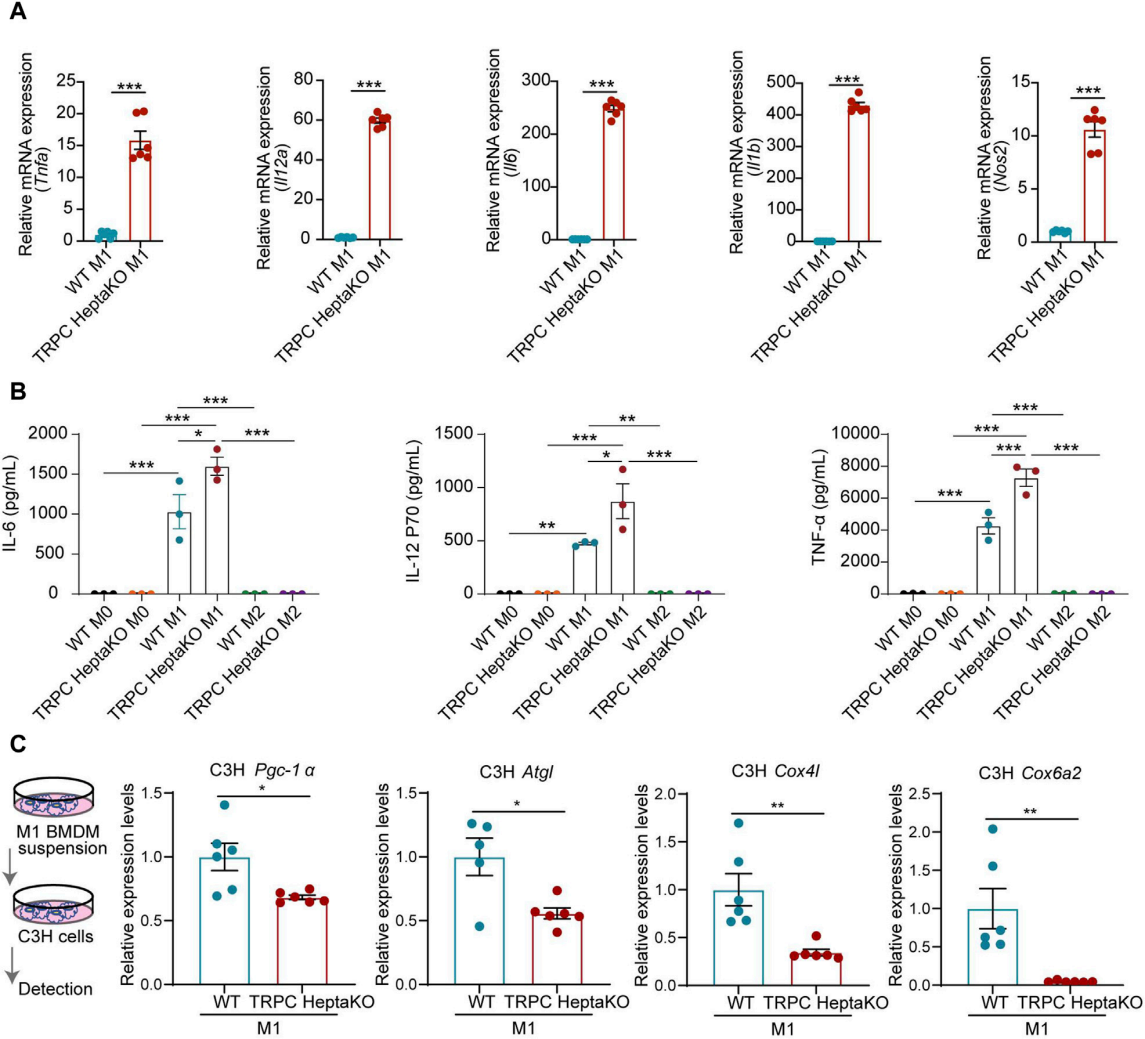
Macrophages from TRPC HeptaKO mice exhibit M1 macrophage polarization and inhibit adipose tissue thermogenesis. **(A)** RT-PCR results of *Il6*, *Nos2*, *Il12α*, and *Tnfα* in BMDMs under LPS/IFN-γ stimulation (n = 4–6). **(B)** The expression levels of IL-1β, IL-6, IL-12p70, and TNF-α from supernatant of BMDMs under LPS/IFN-γ or IL-4 stimulation which were measured by ELISA (n = 3). **(C)** C3H cells were treated with the suspension of M1-BMDM for 24 h. Left: Schematic illustration. Right: The relative expression levels of fatty acid regulation genes in C3H cells (n = 5–6). In Figure 5B, One-way ANOVA was used, whereas for the other figures, Two-tailed Student’s t-test was employed. *p < 0.05, **p < 0.01, ***p < 0.001.

Given that lacking all seven TRPC proteins was associated with a heightened abundance of M1-type macrophages in iWAT, we postulated that TRPC deletion might favor the polarization of macrophages towards the M1 phenotype. To test this hypothesis, we isolated BMDMs from TRPC HeptaKO and WT mice, subsequently treating them with LPS/IFN-γ or IL-4 to stimulate M1 or M2 polarization, respectively. RT-PCR results revealed that compared with WT M1 macrophages, TRPC HeptaKO M1 macrophages dramatically upregulated the expression of M1 macrophage makers including *Il6*, *Il12α*, *Tnfα*, and *Nos2* mRNA (Figure 5A). Moreover, M1 macrophages, not M2 macrophages, from TRPC HeptaKO mice also markedly increased the secretion of proinflammatory cytokines including IL-6, IL-12 P70, and TNF-α (Figure 5B). Furthermore, we treated adipocytes C3H with supernatants from M1 macrophages and discovered that culture media from TRPC-deficient BMDMs significantly suppressed the level of the thermogenic gene *UCP1*, decreased the levels of lipid degradation-related gene *Agtl*, and attenuated the level of mitochondrial viability-related enzymes *Cox4i1*, *Cox6a2* in C3H cells (Figure 5C). Altogether, the absence of all TRPC channels promoted macrophage polarization toward the M1 type, promotes inflammatory factor secretion, which in turn accelerate lipid synthesis and inhibit lipid degradation.

### 3.5 Deletion of all seven TRPC proteins disrupts the gut microbiota composition in mice

**FIGURE 6:**
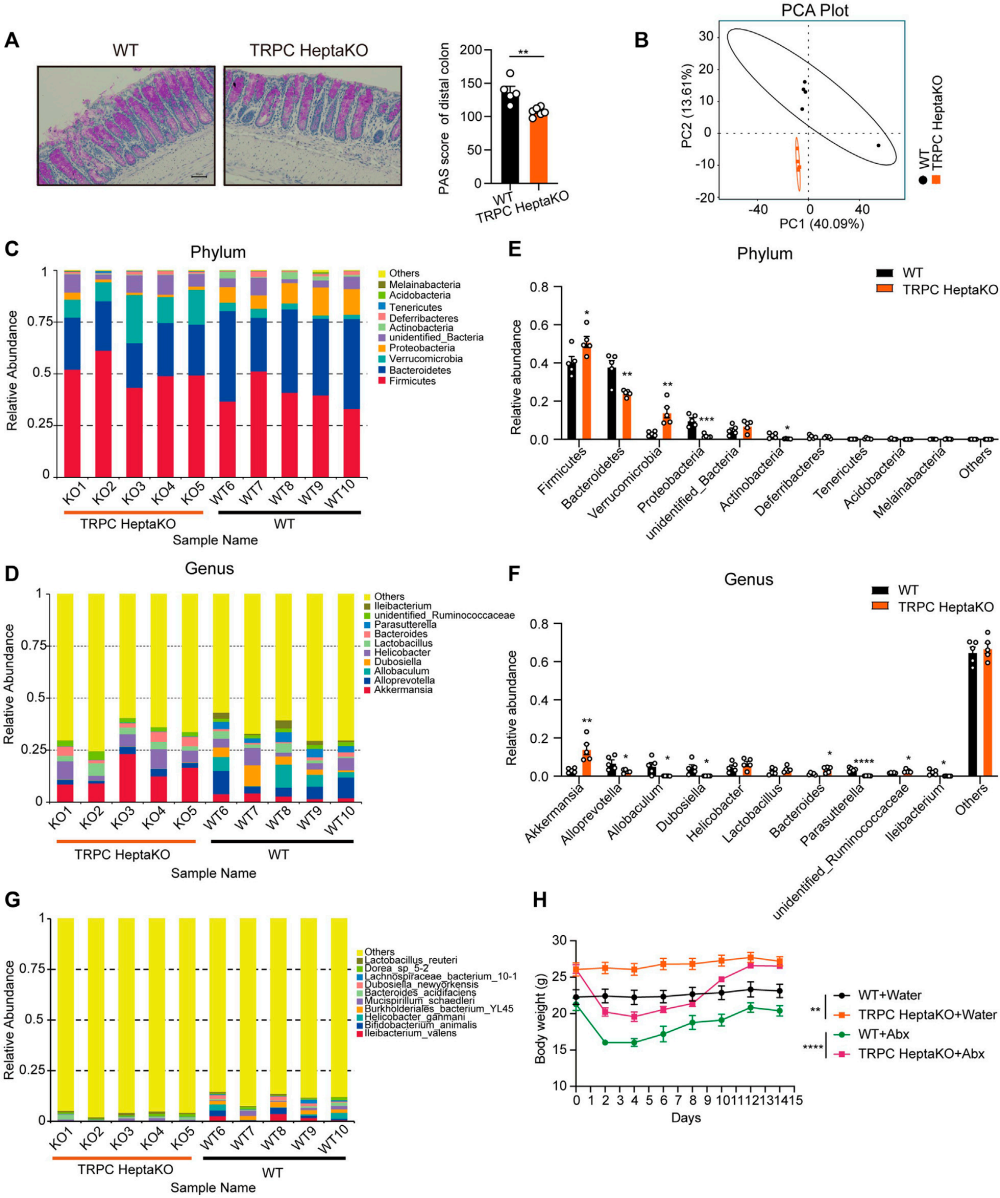
Lacking all seven TRPC proteins results in gut microbiota dysbiosis. **(A)** Representative PAS-stained microscopic images (Left) and quantification of PAS^+^ goblet cells in colonic tissues of WT and TRPC HeptaKO mice (Right). Scale bar: 50 μm. (n = 5–6). **(B)** PCA analysis (beta diversity) of gut microbiota in WT and TRPC HeptaKO mice (n = 5). **(C, D)** The composition and relative abundance of gut microbiota at phylum level and genus level in WT and TRPC HeptaKO mice (n = 5). **(E, F)** Differentially bacteria between TRPC HeptaKO and WT group at phylum and genus (n = 5). **(G)** Species richness and diversity of gut microbiota in WT and TRPC HeptaKO mice (n = 5). **(H)** The body weight of WT and TRPC HeptaKO mice that were treated with Abx or water (n = 4–6). In Figure 6H, Two-way ANOVA test was used, whereas for the other figures, Two-tailed Student’s t-test was employed. *p < 0.05, **p < 0.01, ***p < 0.001.

Goblet cells within the colon secrete mucin two and play a pivotal role in maintaining gut microbiota homeostasis (Johansson, 2012; Birchenough et al., 2016). Given that PAS^+^ goblet cells were notably depleted in TRPC HeptaKO mice under standard conditions (Figure 6A), we performed 16S rRNA analysis in colon content, revealing significant discrepancies in the gut microbiota composition between TRPC HeptaKO and WT mice at both phylum and genus levels (Figures 6B–D). At the phylum level, it was observed that TRPC HeptaKO mice manifested a substantial decrease in the relative abundance of *Bacteroidetes*, *Proteobacteria*, and *Actinobacteria*, while exhibiting a corresponding increase in *Firmicutes* and *Verrucomicrobia* (Figure 6E). Consistent with earlier findings, the gut microbiota profile of obese humans or mice typically presents with a higher *Firmicutes/Bacteroidetes* ratio (Ley et al., 2005; Turnbaugh et al., 2006; Turnbaugh et al., 2008; Riva et al., 2016). Notably, the abundance of *Bacteroidetes*, inclusive of the genus *Alloprevotella*, was notably reduced in TRPC HeptaKO mice. Within the *Proteobacteria* phylum, the genus *Parasutterella* was found to be less prevalent in TRPC HeptaKO mice. Intriguingly, the *Verrucomicrobia* phylum, which includes the mucin-degrading bacterium *Akkermansia*, showed a marked elevation in TRPC HeptaKO mice. Furthermore, within the *Firmicutes* phylum, we detected a significant increase in unidentified members of the *Ruminococcaceae* family in TRPC HeptaKO mice, whereas genera such as *Allobaculum*, *Dubosiella*, and *Ileibacterium* were conversely decreased (Figure 6F). Although the 16S rRNA sequencing technology was incompletely sequenced at the species level, our analysis still discerned substantial differences between the gut microbiota compositions of the two groups at this level (Figure 6G). Taken together, these results demonstrate that the gut microbiota is perturbed in TRPC HeptaKO mice. To explore whether the deletion of TRPC promotes obesity development through alterations in gut microbiota, we provided mice with *ad libitum* access to Abx or control water for a period of 2 weeks, subsequently monitoring their body weight. Administration of Abx did not significantly affect the body weight of mice (Figure 6H), indicating that TRPC deletion-induced obesity alters the composition of the gut microbiota of mice, and in turn, the gut microbiota does not mediate TRPC knockout-induced obesity.

### 3.6 Deletion of all seven TRPC proteins accelerates the onset of AOM/DSS-induced CRC

**FIGURE 7:**
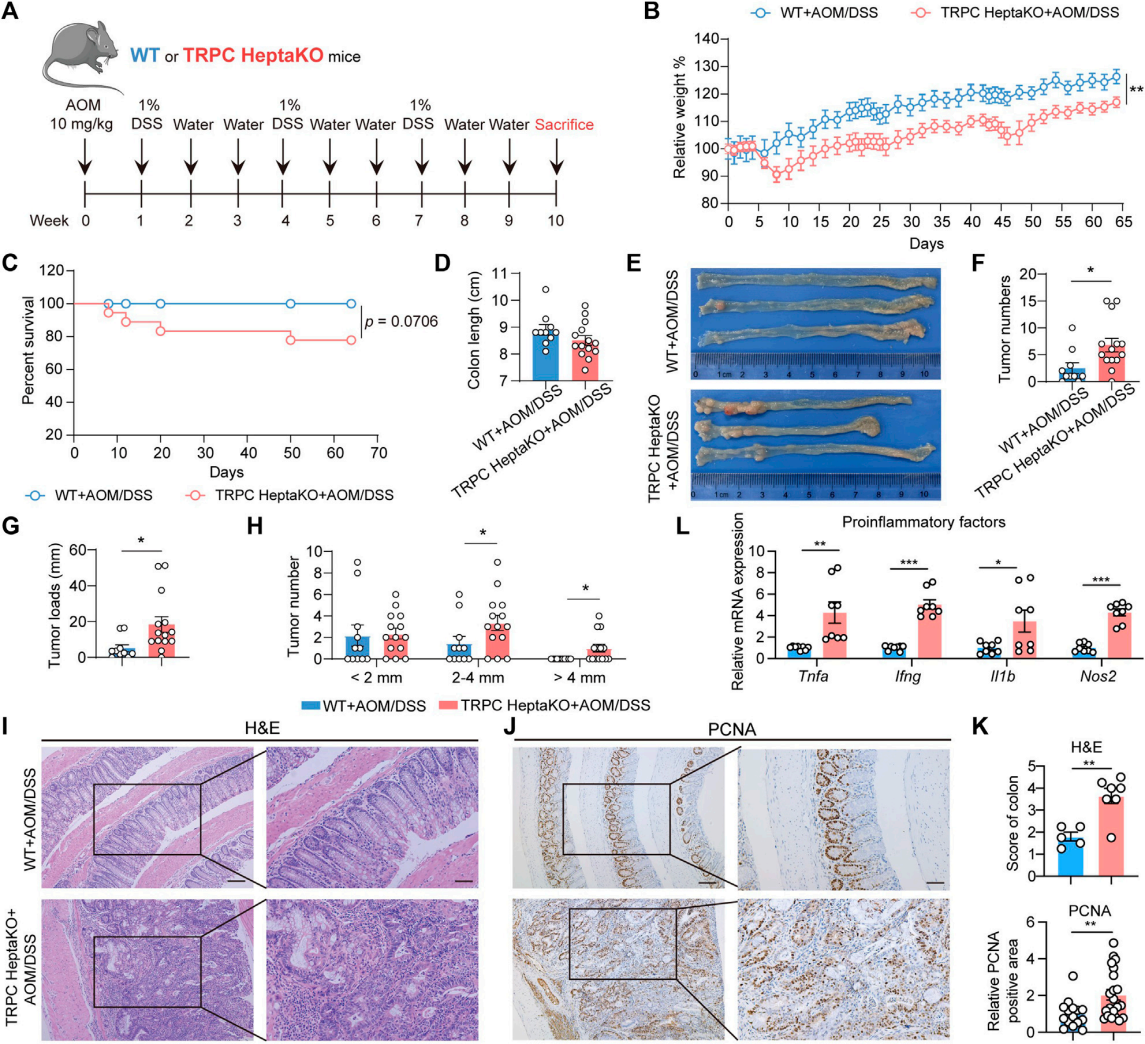
Lacking all seven TRPC proteins facilitates the development of AOM/DSS-induced CRC. **(A)** Schematic illustration of AOM/DSS-induced CRC strategy. **(B, C)** Body weight and survival rate of AOM/DSS-induced WT and TRPC HeptaKO mice (n = 11–19). **(D)** Colon length (n = 10–14). **(E)** Representative images of the colon in each group. **(F, G)** Tumor number and tumor loads (tumor loads = the sum of diameters of all tumors in colon, n = 10–14). **(H)** Tumor number (n = 10–14). **(I–K)** Representative H&E stained and PCNA-images and pathological scores in the colon tissues (n = 5–7, PCNA pathological scores: three fields/slice). **(L)** *Tnfa*, *Ifng, Il1b*, and *Nos2* mRNA levels in colorectal tissues of AOM/DSS-induced WT and TRPC HeptaKO mice (n = 8). Two-tailed Student’s t-test was used to determine statistical significance between two groups. *p < 0.05, **p < 0.01, ***p < 0.001.

Extensive research has underscored that individuals with disrupted gut microbiota profiles are at a heightened risk of developing CRC compared to healthy individuals (Wong and Yu, 2023). Consequently, to assess the effect of the absence of all seven TRPC proteins on CRC development, we constructed an AOM/DSS-induced CRC model using TRPC HeptaKO mice and WT controls (Figure 7A). As anticipated, the deletion of all seven TRPC channels led to a reduction in body weight and a decrease in survival rates in mice subjected to AOM/DSS treatment (Figures 7B, C). Despite not affecting the overall colon length in these mice (Figure 7D), TRPC HeptaKO mice displayed a greater number and burden of tumors in their colonic tissues relative to WT mice (Figures 7E–G). Significantly, the incidence of tumors measuring over 2 mm in diameter was notably augmented in TRPC HeptaKO mice (Figure 7H). Moreover, TRPC HeptaKO mice exhibited intensified colonic inflammation, dysplasia, adenocarcinoma formation, mucosal damage, and cellular proliferation within the colon tissues when compared to WT mice (Figures 7I–K). It is well-documented that proinflammatory factors play a central role in CRC progression (Vendramini-Costa and Carvalho, 2012). Thus, we proceeded to evaluate the expression of these proinflammatory factors in colon tissues. Confirming our hypothesis, the expression levels of several key proinflammatory factors, including *Tnfa*, *Ifng*, *Il1b*, and *Nos2*, were considerably elevated in the colon tissues of AOM/DSS-treated TRPC HeptaKO mice (Figure 7L). In summary, our findings collectively indicate that the absence of all seven TRPC channels expedites the progression of AOM/DSS-induced CRC.

## 4 Discussion

Multiple TRPC channels play a pivotal role in the progression of obesity and its related complications, yet the underlying mechanism remains elusive. Notably, TRPC inhibitors have gained widespread usage in clinical or preclinical settings for treating diverse diseases, including cardiovascular diseases and diabetic nephropathy. Nevertheless, reports on the potential adverse effects of these inhibitors, particularly in the context of obesity and obesity-induced CRC, are scant. In this study, we have uncovered a crucial function of TRPC channels in mediating obesity and obesity-associated CRC. Our findings reveal that TRPC deficiency leads to a surge in pro-inflammatory macrophages and inflammatory factors within adipose tissue, triggering lipid accumulation and exacerbating obesity. Furthermore, this deficiency-induced obesity profoundly disrupts the gut microbiota, thereby fostering CRC progression. Collectively, this study sheds light on the role and mechanism of TRPC deficiency in obesity and its related CRC complications, offering a potential theoretical framework for averting adverse effects associated with TRPC inhibitors.

Considering the broad expression of TRPC channels across numerous tissues, their critical role in Ca^2+^ influx through intracellular stores and/or receptor-operated pathways, and their profound influence on diverse physiological functions across various organ systems, TRPCs have received considerable attention as potential therapeutic targets for various diseases. Due to the similarities between molecules within the TRPC subfamilies, which lead to their propensity for heteromerization, such as the tendency for TRPC3/6/7 to form heteromers (Liu et al., 2024), the selectivity of some current TRPC inhibitors remains to be improved. For instance, Pico145 (also known as C31, HC-608), a xanthine derivative, can simultaneously inhibit TRPC1/4/5 channels (Rubaiy et al., 2017). Given the molecular similarities among TRPC channel subfamilies and the non-selectivity of related inhibitors, we have utilized TRPC whole-family knockout mice to investigate phenotypic and mechanistic outcomes following TRPC deletion, thereby providing potential intervention for adverse reactions that may arise from TRPC inhibitors.

TRP channels are non-selective calcium-permeable cation channels mainly located on the plasma membrane. These channels have been implicated in diverse physiological processes, including thermogenesis in adipose tissue, energy metabolism, and body weight regulation. Notably, calcium influx mediated by TRPV1, TRPV2, TRPV4, TRPM8, and TRPC1 has been reported to modulate the expression of thermogenic genes in adipocytes, such as UCP1 and PGC-1α, leading to enhanced thermogenesis (Sun et al., 2021). Conversely, the inhibition of these channels blocks calcium influx, thereby suppressing adipocyte thermogenesis and potentially promoting obesity. For TRPC channels, TRPC3/6/9 are also typical channels that regulate calcium influx (Fan et al., 2018; Guo et al., 2022). In this study, we observed a significant reduction in the levels of thermogenic molecules, including UCP1, PGC-1α, *Pparg*, *Prdm16*, and *Cidea*, in the absence of TRPC. This imbalance between energy expenditure and intake contributes to adiposity and, over time, may lead to the development of obesity. While our current investigation primarily focused on the suppression of thermogenic gene expression in adipose tissue due to local inflammation in the absence of TRPC, further exploration is needed to determine whether this effect is influenced by the blockade of calcium influx.

Multiple studies have consistently demonstrated that chronic inflammation plays a pivotal role in the onset and exacerbation of obesity and its related complications. Adipose tissue macrophages (ATMs), which account for about 5% of the stromal vascular fraction (SVF) within lean body states but surge to approximately 50% in obesity, are widely acknowledged as instrumental drivers of obesity-related inflammation (Weisberg et al., 2003). Macrophages typically fall into two categories: pro-inflammatory (M1 type) or anti-inflammatory (M2 type). In an obese condition, there’s a tendency for macrophages to polarize towards the M1 phenotype, thereby accelerating inflammation in adipose tissues, promoting lipid accumulation, and contributing to systemic insulin resistance. Conversely, a shift from M1 to M2 polarization is linked with attenuated inflammation, decreased body weight, enhanced insulin sensitivity, and the browning of white adipose tissue (Chavakis et al., 2023). In our research, we observed that *Il6* and macrophage-related chemokines *Ccl4*, *Ccl5*, and *Cxcl9* were notably elevated in the adipose tissue of TRPC HeptaKO mice, indicating a potential rise in pro-inflammatory macrophages, which was further validated through flow cytometry analysis. Additionally, we found that the deletion of TRPC led to a significant increase in the secretion of pro-inflammatory factors such as IL-6, IL-12, and TNF-α specifically from M1-type BMDMs. To further investigate the potential impact of pro-inflammatory macrophages on adipocyte thermogenesis, we subjected adipocytes to treatment with conditioned media from BMDMs. The results revealed a substantial decrease in the expression of thermogenic molecules within adipocytes, thus suggesting that TRPC deficiency-induced macrophage inflammation suppresses adipocyte thermogenesis and encourages lipid accumulation. This study did not directly address the underlying mechanism by which the absence of TRPC leads to an augmented macrophage presence in adipose tissue, a question that remains open and requires further investigation.

Obesity frequently coexists with numerous complications, including common ailments like diabetes and cardiovascular diseases, as well as a close association with the incidence of CRC. In Europe, an estimated 11% of CRC cases can be attributed to excess weight and obesity (Bardou et al., 2022). Epidemiological evidence underscores that obesity heightens the risk of developing colon cancer in men by 30%–70% (Bardou et al., 2022). A meta-analysis has demonstrated that for every 5 kg/m^2^ rise in Body Mass Index (BMI), the likelihood of colorectal adenomas increases by a notable 20% (Dai et al., 2007). Research indicates that obesity can alter the gut microbiome composition, fostering the progression of CRC. High-fat diet (HFD)-fed mice experience a marked shift in their gut microbiota, characterized by an increase in potentially pathogenic bacteria such as *Alistipes sp*. *Marseille-P5997* and *Alistipes* sp. *5CPEGH6*, a decline in beneficial species like *Parabacteroides distasonis*, and a weakened intestinal barrier function, all of which contribute to CRC development (Yang et al., 2022). In our study, we observed that TRPC deletion resulted in a decrease in the number of goblet cells in the colon, hinting at a possible alteration in the gut microbiota of the mice. Through 16S rRNA sequencing, we indeed detected significant disparities in the gut microbiota composition between WT and TRPC-KO mice, with a higher *Firmicutes*/*Bacteroidetes* ratio in the TRPC HeptaKO group, which aligns with prior findings. To further probe the connection between TRPC-mediated obesity and changes in gut microbiota, we administered Abx to both WT and TRPC HeptaKO mice to eradicate their gut microbiota. The outcome revealed that the body weights of both groups remained unaltered, implying that the modification in gut microbiota was a consequence of obesity in the TRPC HeptaKO mice. Moreover, the heightened susceptibility of TRPC HeptaKO mice to CRC might be linked to the shifted gut microbiota composition, exemplified by a significant elevation in *Bacteroides*, a commonly reported cancer-promoting bacterium (Sun et al., 2023), within the colons of TRPC HeptaKO mice. However, this study did not delve into the specific mechanisms behind CRC development due to these microbiota alterations; this remains an area requiring further exploration.

In summary, this study focuses on investigating the impact and preliminary mechanisms of the complete family deletion of TRPC channels on obesity and its associated complication CRC, elucidating potential mechanisms underlying adverse reactions to TRPC non-selective inhibitors, with the aim to provide a theoretical foundation for future clinical interventions to mitigate adverse effects such as the inhibition of pro-inflammatory macrophage polarization upon TRPC blockers. However, there remain several unresolved issues in this research, including how TRPC deficiency promotes pro-inflammatory macrophage polarization, how TRPC knockout-induced gut microbiota dysbiosis exacerbates CRC development, and whether similar phenotypes would occur with single TRPC channel knockouts, all of which are critical questions to be further explored in our subsequent studies.

## 5 Conclusion

In conclusion, our research has disclosed that the absence of TRPC culminates in the polarization of pro-inflammatory macrophages, which in turn drives obesity, disrupts the gut microbiota balance, and exacerbates CRC. This study elucidates the diseases and potential mechanisms that may occur when the cation channels TRPC are absent or blocked, increasing our understanding of the physiopathological functions of TRPC channels and providing a theoretical basis for preventing potential adverse effects.

## Data Availability

All data relevant to the study are included in the article/Supplementary Material. The datasets from the 16S rRNA gene sequencing have been deposited in the National Center for Biotechnology Information’s (NCBI) public BioProject repository under the accession number PRJNA906746, which can be accessed via this link: https://www.ncbi.nlm.nih.gov/bioproject/?term=PRJNA906746. Any additional raw data supporting the conclusion of this article will be made available by the authors, without undue reservation.
