# Supplementary material for: TRPC absence induces pro-inflammatory macrophage polarization to promote obesity and exacerbate colorectal cancer
Source: Front Pharmacol. 2024 May 21;15:1392328. doi: 10.3389/fphar.2024.1392328 (PMC11148282; doi:10.3389/fphar.2024.1392328)
Supplement: Supplementary file 1 [file Table1.DOCX]

**Supplementary Table 1**. The primer sequences for RT-PCR.

| Gene | | Sequence (5’to 3’) |
| --- | --- | --- |
| *Pparγ* | F | TCGCTGATGCACTGCCTATG |
|  | R | GAGAGGTCCACAGAGCTGATT |
| *Prdm16* | F | CCACCAGCGAGGACTTCAC |
|  | R | GGAGGACTCTCGTAGCTCGAA |
| *Cidea* | F | ATCACAACTGGCCTGGTTACG |
|  | R | TACTACCCGGTGTCCATTTCT |
| *Il1b* | F | CAATGGACAGAATATCAAC |
|  | R | ACAGGACAGGTATAGATT |
| *Il6* | F | TAGTCCTTCCTACCCCAATTTCC |
|  | R | TTGGTCCTTAGCCACTCCTTC |
| *Ccl4* | F | TTCCTGCTGTTTCTCTTACACCT |
|  | R | CTGTCTGCCTCTTTTGGTCAG |
| *Ccl5* | F | GCTGCTTTGCCTACCTCTCC |
|  | R | TCGAGTGACAAACACGACTGC |
| *Cxcl9* | F | GGAGTTCGAGGAACCCTAGTG |
|  | R | GGGATTTGTAGTGGATCGTGC |
| *Tnfa* | F | CTGAACTTCGGGGTGATCGG |
|  | R | GGCTTGTCACTCGAATTTTGAGA |
| *Il-12a* | F | ACATCTGCTGCTCCACAAG |
|  | R | GGTGCTTCACACTTCAGGAA |
| *Nos2* | F | ACATCGACCCGTCCACAGTAT |
|  | R | CAGAGGGGTAGGCTTGTCTC |
| *Pgc1α* | F | TATGGAGTGACATAGAGTGTGCT |
|  | R | CCACTTCAATCCACCCAGAAAG |
| *Atgl* | F | ATGTTCCCGAGGGAGACCAA |
|  | R | GAGGCTCCGTAGATGTGAGTG |
| *Cox4il* | F | ATTGGCAAGAGAGCCATTTCTAC |
|  | R | CACGCCGATCAGCGTAAGT |
| *Cox6a2* | F | CCAGAGTTCATCCCGTATCACC |
|  | R | GATTGTGGAAAAGCGTGTGGT |
| *Actin* | F | ACCACACCTTCTACAATGAG |
|  | R | ACGACCAGAGGCATACAG |
